# Magneto-Optical Surface Plasmon Resonance Multi-Spot Assay for Identification and Quantification of Gaseous Compounds at Room Temperature

**DOI:** 10.3390/s26144537

**Published:** 2026-07-17

**Authors:** Sorin David, Cristina Polonschii, Elena Gabriela Cocos-Barbu, Dumitru Bratu, Eugen Gheorghiu

**Affiliations:** 1 International Centre of Biodynamics, Intrarea Portocalelor 1B, Sector 6, 060101 Bucharest, Romania; cpolonschii@biodyn.ro (C.P.); dbratu@biodyn.ro (D.B.); egheorghiu@biodyn.ro (E.G.); 2 Faculty of Medical Engineering, University Politehnica of Bucharest, Splaiul Independenţei nr. 313, Sector 6, 060042 Bucharest, Romania; cocosgabriela1@gmail.com; 3 Faculty of Biology, University of Bucharest, Blvd. Regina Elisabeta, 030018 Bucharest, Romania

**Keywords:** volatile organic compounds, magneto-optical surface plasmon resonance, surface plasmon resonance, gas sensing, multi-spot assay, magneto-plasmonic sensors, principal component analysis, shrinkage linear discriminant analysis

## Abstract

Rapid room-temperature identification of gases and volatile organic compounds remains challenging for compact sensing platforms, particularly when chemically related analytes must be discriminated using accessible sensing materials. In this work, we evaluate whether magneto-optical surface plasmon resonance (MOSPR), combined with multi-spot sensing and conventional SPR readout from the same chip, can provide complementary response features for improved gas/VOC discrimination. The sensing spots are made from accessible chemicals and nanoparticles with plasmonic and magnetic properties. The sensor chip consists of a multilayer structure of metallic materials with both plasmonic and magnetic properties featuring enhanced sensitivity and stability. Measurements are made using a custom-built MOSPR instrument at relevant analyte concentrations. Analyte-specific sensor channels were selected for concentration-dependent calibration while the complete multivariate data were first explored using principal component analysis for supervised analyte classification. The combined 16-feature MOSPR/SPR model achieved an overall accuracy of 88.3% and a balanced accuracy of 87.6% under leave-one-concentration-block-out cross-validation compared with 66.2% and 65.7%, respectively, for the SPR measurement alone. These results show that MOSPR provides response information that encompasses and extends that obtained from conventional SPR measurements, thereby improving analyte discrimination. The proposed approach may provide a basis for future environmental monitoring and industrial process control, including real-time monitoring of harmful gaseous emissions pending further validation under application-specific conditions.

## 1. Introduction

Volatile organic compounds (VOCs) are organic chemical compounds (gases or vapors) emitted from certain solids or liquids. VOCs include aromatic hydrocarbons, aliphatic hydrocarbons, aldehydes, ketones, ethers, acids and alcohols, with diverse functional groups (halogens, oxygen, sulphur, nitrogen or phosphorus, excluding carbon oxides and carbonates) [1]. In this work, we focus on the detection of some important VOCs like methane, acetone, isopropanol, and ethanol. Methane, a greenhouse gas, is usually emitted from natural sources (wetlands, livestock, and geological processes) and human activities (agriculture, fossil fuel extraction, and waste management). Besides its contributions to global warming and climate change, methane is a precursor to ground-level ozone formation and contributes to air pollution, posing risks to human health and ecosystems [2]. As a commonly encountered solvent, acetone is an important VOC of indoor and outdoor air pollution, resulting from industrial emissions, vehicle exhaust, or household products. In industrial applications, accurate monitoring of acetone concentrations is essential for process control while ensuring workplace safety. Moreover, acetone is a biomarker for certain metabolic disorders such as diabetes and ketosis, making its detection highly relevant in clinical diagnostics [3]. Isopropanol is another VOC with wide-ranging industrial, medical, and household applications. In medical diagnostics, the detection of isopropanol in biological samples aids in diagnosing some metabolic disorders and in monitoring ethanol metabolism [4]. Ethanol is a chemical commonly found in alcoholic beverages, pharmaceuticals, cosmetics, and industrial products. The importance of detecting ethanol as a volatile organic compound lies in its widespread applications across various industries and its potential impact on health and safety in environmental monitoring, research, and forensic science [5]. This analyte set was selected to cover both environmentally and industrially relevant VOCs and gases with different chemical and physical properties. Methane was included as a small non-polar combustible gas and greenhouse gas; acetone, ethanol, and isopropanol were selected as common oxygenated VOCs with relevance to indoor air quality, industrial safety, and process monitoring. CO_2_ and N_2_ were included as reference gaseous components of air, allowing the response of the MOSPR/SPR multi-spot platform to be evaluated not only for VOC vapors but also for non-VOC gases present in atmospheric or process-monitoring contexts.

Detection, identification, and classification of VOCs are currently done using gas sensors and electronic noses [6]. Electronic noses generally consist of a sampling device, a system of sensors producing a multivariate signal after reversible interaction with the mixture of compounds present in the gaseous sample, and a system for processing the multivariate signals for identifying patterns related to the presence, type, and intensity of target analytes to discriminate among various contamination sources. The sensing system usually comprises an array of transducers including chemoresistors, such as metal-oxide-semiconductor (MOS) sensors, metal-oxide-semiconductor field-effect transistors (MOSFET), and composite conducting polymer (CCP) sensors; polymer-functionalized quartz crystal microbalance sensors (QCM); surface acoustic wave (SAW) sensors; polymer-coated fiber-optic evanescent wave (FOEW) sensors; polymer-functionalized micro-electromechanical system (MEMS) cantilever sensors; and surface plasmon resonance (SPR) sensors [7]. Signals of electronic noses are analyzed to identify changes in the composition of sampled air, related to gaseous emissions [6]. Recent developments in gas and VOC sensing have increasingly moved from single-sensor detection toward sensor-array and electronic-nose concepts, in which partially selective sensing elements generate multidimensional response patterns that are subsequently interpreted using chemometric or machine-learning methods [8]. This approach is particularly useful for VOC discrimination, because chemically related analytes may produce overlapping responses on individual sensing layers. Recent reviews emphasize that the performance of electronic-nose systems depends not only on the sensing material and transducer, but also on the signal-processing pipeline used for pattern recognition, drift reduction, and concentration estimation. In this context, multivariate methods such as PCA, LDA, support vector machines, random forests, and neural-network-based approaches have been used for gas classification and, in more advanced cases, mixed-gas analysis. Recent work has also shown that dynamic or local neural-network models can be used for quantitative analysis of mixed gases, although such approaches generally require larger training datasets and dedicated mixture-calibration experiments [9]. Currently, most electronic noses applied for environmental monitoring are based on instruments designed for applications in controlled conditions [6]. Environmental monitoring for relatively long periods (e.g., several months to one year) requires checks and periodic recalibrations for the appropriate functioning of multi-sensor systems such as electronic noses. At present, instrumental approaches and procedures for identifying the presence of odorant mixtures with high temporal resolution are not standardized, and studies highlighting operational pitfalls and solutions are required to define successful strategies for characterizing complex situations in open systems [10]. Optical gas sensors based on SPR or LSPR offer an alternative route to VOC monitoring by detecting refractive-index or optical-property changes induced by adsorption or interaction of gaseous compounds with functional sensing layers [11]. Recent SPR-based and Fabry–Pérot optical sensors functionalized with materials such as metal–organic frameworks have demonstrated VOC detection at room temperature, while plasmonic nanostructures have been used to improve optical gas-sensing response. Magneto-optical SPR further extends conventional SPR by combining plasmonic excitation with transverse magneto-optical Kerr modulation in noble-metal/ferromagnetic structures. This modulation can enhance the sensitivity of SPR-based measurements and provide additional response features compared with conventional SPR alone. In this work, we aim to improve the capabilities for air quality monitoring by developing a fast, sensitive, and reliable device for the detection and monitoring of outdoor and indoor VOCs based on Magneto-Optical Surface Plasmon Resonance [12] (MOSPR), a magnetically augmented SPR [13] technique. So far, this method has been applied to increase the sensitivity of SPR by ~ two orders of magnitude [12,14] in gas [15] or liquid [16]. The approach is based on the transverse magneto-optical Kerr effect (TMOKE) and involves the use of advanced materials featuring magneto-plasmonic properties. TMOKE takes place when applying a magnetic field perpendicular to the plane of propagation of the incident p-polarized light and in the plane of a thin film with magneto-optical properties, causing changes in the intensity of the reflected light [17]. The combination of TMOKE with plasmonic effects has established a new detection configuration, namely the MOSPR technique [18]. In a TMOKE setup, plasmons are excited, resulting in a modulation of the reflected intensity curve. The recorded MOSPR response presents increased sensitivity to minute changes in the refractive index within the close vicinity of the sensor chip. In MOSPR, plasmonic excitation is combined with transverse magneto-optical Kerr modulation in noble-metal/ferromagnetic multilayers or alloys. The magnetic component enables modulation of the reflected intensity under an alternating transverse magnetic field, while the plasmonic metal supports the surface plasmon resonance. The magnitude and stability of the MOSPR response depend strongly on the composition and thickness of the magneto-plasmonic film, since excessive ferromagnetic content may damp the SPR curve, whereas insufficient magnetic material may reduce the magneto-optical modulation. A further limitation of this technique is, however, the reduced stability of the multilayered chip with magneto-plasmonic properties [19]. A method for improving the stability of chips with magneto-plasmonic properties using a noble-metal/ferromagnetic-metal alloy was proposed and successfully applied by our group for monitoring real-time antigen–antibody interactions [19].

**Figure 1:**
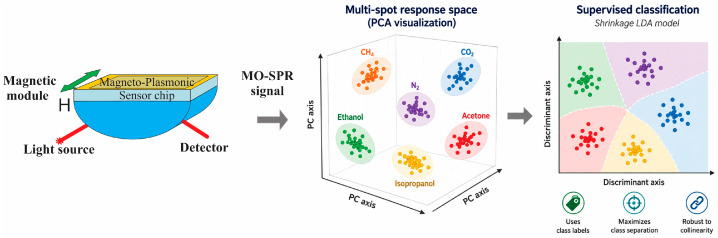
MOSPR gas detection with analysis concept.

This method and the corresponding measurement configuration [19,20] open new possibilities for MOSPR applications by improving contrast and resolution in multi-spot analysis. The present work builds on this framework by adapting the previously developed Au-Co magneto-plasmonic chip architecture and MOSPR readout to multi-spot gas/VOC measurements. The sensing area consists of multiple spots of gas-sensitive polymers [21,22] and metallic oxides [23] and magneto-plasmonic nanoparticles. Rather than relying on a single highly selective receptor, the assay exploits the differential response pattern generated by several functionalized spots. We use a measurement device based on modular benchtop MOSPR equipment developed by our team [19] combined with a portable SPR platform. The collected data were first explored using PCA as an unsupervised visualization method and were subsequently analyzed using shrinkage LDA as a supervised and regularized classifier suitable for datasets with correlated features and a limited number of independent experimental blocks. The analysis of the interaction of several spots with individual VOCs provides a specific “fingerprint” for each VOC. Concentrations were estimated using the analyte-specific responses. The assay concept is presented in Figure 1. In the present proof-of-concept study, we focus on individual gas/VOC exposures in an air background as a first step toward establishing analyte-specific response fingerprints of the MOSPR/SPR multi-spot platform. Extension to calibrated binary and multicomponent gas mixtures, together with dedicated mixed-gas modeling approaches, will be addressed in future work.

## 2. Materials and Methods

**Figure 2:**
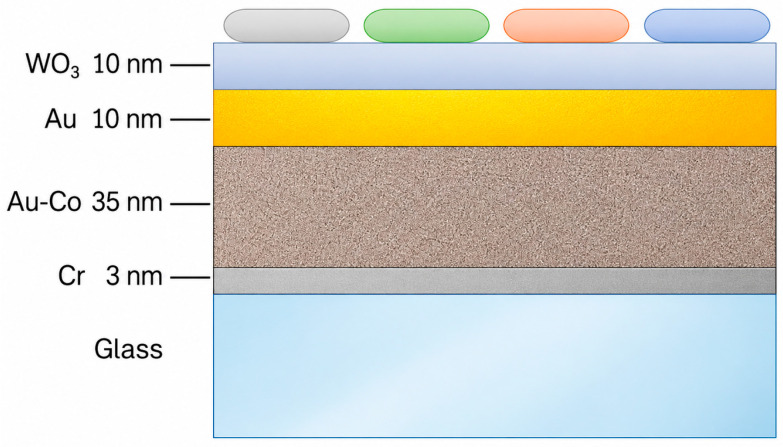
MOSPR gas-sensing chip architecture and sensing-spot layout. Schematic representation of the multilayer magneto-plasmonic chip, showing the glass substrate, 3 nm Cr adhesion layer, 35 nm Au-Co magneto-plasmonic alloy layer, 10 nm Au capping layer, 10 nm WO_3_ layer, and deposited gas-sensitive spots.

The magneto-plasmonic chips are prepared by thermal evaporation using physical vapor deposition (PVD75, Kurt J. Lesker, Jefferson Hills, PA, USA). The BK7 glass slides (MGM, Bucharest, Romania) were cleaned, and 3 nm of chromium was deposited to promote the adhesion of the following layers. A 35 nm Au-Co layer alloy (90:10 *w*/*w*) was then deposited as the magneto-plasmonic film, followed by a 10 nm Au layer to improve the chemical stability. This sensor configuration has the SPR minimum in gaseous environments (n~1) at approximately 45°. To shift the SPR minimum in the measurement range of the instrument, a 10 nm layer of WO_3_ is deposited on top of the gold. After preparation, the sensor chip surface is hydrophilized by exposure to UV-Ozone plasma using a PSD-UV instrument(Novascan Technologies Inc., Ames, IA, USA) to improve the wetting properties for the subsequent spot deposition. The sensitive spots are based on a dextran gel matrix (DEX) in which we embedded, in various proportions, magneto-plasmonic (Au, Fe_2_O_3_) and gas-sensitive materials (TiO_2_ and polyaniline). We developed gas-sensitive spots using materials that are accessible and easy to prepare. We realized four sensitive spots based on various nanoparticles: polyaniline, TiO_2_, gold and magnetic ones. Polyaniline (PAN) is a conductive organic polymer with sensing applications based on its ability to change its optical and electrical properties in various oxidation states, in a scale dependent on the presence and concentration of selected gases. It was used in powder form and resuspended in distilled water at a concentration of 1 mg/mL [24]. TiO_2_ is a compound with photocatalytic properties that interact with various VOCs and nitrogen oxides. TiO_2_ nanoparticles (with a diameter of 100 nm) in aqueous dispersion were diluted 1:10 in water. A schematic representation of the multilayer chip structure is presented in Figure 2.

The fabrication and magneto-optical characterization of the Au-Co alloy magneto-plasmonic chip architecture were previously reported in detail [19,20]. In those studies, the Au-Co alloy film was characterized by SEM-EDX composition analysis, theoretical and experimental SPR curves, TMOKE response under an alternating transverse magnetic field, signal-to-noise behavior, and comparison between MOSPR and classical SPR. In the present work, this previously validated platform was further adapted by adding the WO_3_ layer and multi-spot gas-sensitive coating for gas/VOC measurements.

Magnetic nanoparticles (GMB), coated with gold and stabilized with PEG 3000 (having a diameter of 50 nm) in aqueous dispersion, were diluted 1:10 in water. The above materials were mixed in different proportions (vol:vol) to obtain 4 different chemical compounds: DEX (1% dextran solution in 100 mM NaOH), DEXMBTIO (DEX:GMB:TIO 100:1:1), DEXMB (DEX:GMB 100:1), PANMBTIO (DEX:PAN:GMB:TIO 100:1:1:1). These spots were deposited using a Tecan Cavro Omni Robot (Tecan Männedorf, Switzerland) liquid handling device.

GMB and TiO_2_ nanoparticles were purchased from CD Bioparticles USA; polyaniline (emeraldine base) and dextran sodium salt were purchased from Sigma-Aldrich (St. Louis, MO, USA). Au, Co, Cr (chromium rod) and WO_3_ deposition materials were purchased from Kurt J. Lesker (Jefferson Hills, PA, USA). Unless otherwise stated, all aqueous dilutions were prepared using ultrapure water.

The MOSPR chip is mounted in the measurement device based on a modular benchtop MOSPR equipment integrated into the SPR platform based on a Kretschmann-Raether configuration (sketched in Figure 1). Illumination was provided by an L850P050 laser diode operating at 850 nm (Thorlabs, Newton, NJ, USA) driven by a custom-built power-supply unit. The emitted beam was collimated, expanded, and p-polarized using appropriate optical elements (Thorlabs, Newton, NJ, USA) before being focused onto the center of a custom-built BK7 hemicylindrical coupling prism (MGM, Bucharest, Romania) supporting the MOSPR chip. BK7 was selected to match the refractive index of the glass substrate used for chip fabrication (*n*(850 nm) = 1.5098 ). The optical configuration allowed simultaneous illumination over an angular interval of approximately 10°, centered on the SPR minimum selected by the user. The reflected light was collected using a monochrome Zelux CMOS camera, 1.6 MP, 67 fps at 720 × 540 pixels, USB 3.0 interface (Thorlabs, Newton, NJ, USA), and the reflected intensity was processed as a function of the incidence angle to reconstruct the SPR curve for each selected region of interest. The magneto-plasmonic effect is induced by applying an oscillating transverse magnetic field of a magnitude of 230 Gauss and at a frequency of 1 Hz to the measurement chip and measuring the SPR signal using a MOSPR actuation module. The MOSPR actuation module consisted of two electromagnets (ECAS Electro, Bucharest, Romania) equipped with custom-built magnetic cores and positioned close to the measurement chip to generate a transverse magnetic field. The magnetic field was applied in the plane of the magneto-plasmonic film and perpendicular to the plane of propagation of the incident p-polarized light. The electromagnets were driven by a custom-built control and power-supply unit, which generated the alternating actuation signal used for MOSPR measurements. To limit heating during operation, the electromagnets were coupled to a custom-built cooling system based on Peltier elements (Farnell, Leeds, UK). The alternating magnetic field modulated the reflected intensity, allowing extraction of the MOSPR signal from the amplitude of the magnetically induced oscillations. The setup is used to analyze methane, acetone, ethanol, and isopropanol VOCs, as well as CO_2_ and N_2_ as reference gases. The sensor was kept in ambient air, and the volatile analytes and gases were injected over the sensitive area using a syringe at a flow rate of 100 µL min^−1^ for 10 s. This configuration was used to mimic exposure to target gaseous compounds in an environmental air background. Data were collected and processed using customized software developed in LabVIEW version 8.3 (National Instruments Co., Austin, TX, USA).

Three to five replicate measurements of the selected gases and VOCs were acquired for each analyte-concentration block. The sensor is kept in ambient air and the analytes are injected over the sensitive spots using a syringe. This is done to mimic real-world usage in which the sensor is exposed to contaminants in the environmental atmosphere. The gases (N_2_ and CO_2_) are injected in gas/air mixtures of 25%, 50% and 100%. VOCs are injected and detected at concentrations in the following ranges: acetone 200–1000 ppm, ethanol 50–1000 ppm, isopropanol 50–1000 ppm and methane 500–2000 ppm. Concentrations of the VOCs are validated by performing similar injections over commercial detectors (MQ2 and MQ3, Hanwei Electronics, Zhengzhou, China). During the exposure to analytes, both the magneto-optical and SPR signals are recorded. We analyzed the data in reflectivity and minimum mode. In the reflectivity mode, the reflectivity, *R*, at the most significant angle, i.e., the angle for which the slope of the reflectivity curve is the steepest, is collected. This angle is derived using the method of zero crossing of the derivative applied to the reflectivity curve. Similarly, as in previous works [16], the MO-SPR signal, i.e., Δ*R*/*R*_0_, is extracted by calculating the amplitude of the induced magnetic oscillations, which represents Δ*R* relative to the reflectivity measured without magnetization (derived by averaging the oscillations). The magneto-optical signal is obtained using the formula *cot* (*θ_min_*) ∗ Δ*θ*, where *θ_min_* is the incidence angle for the SPR minimum and Δ*θ* is the angular shift due to magnetic field modulation.

## 3. Results and Discussion

**Figure 3:**
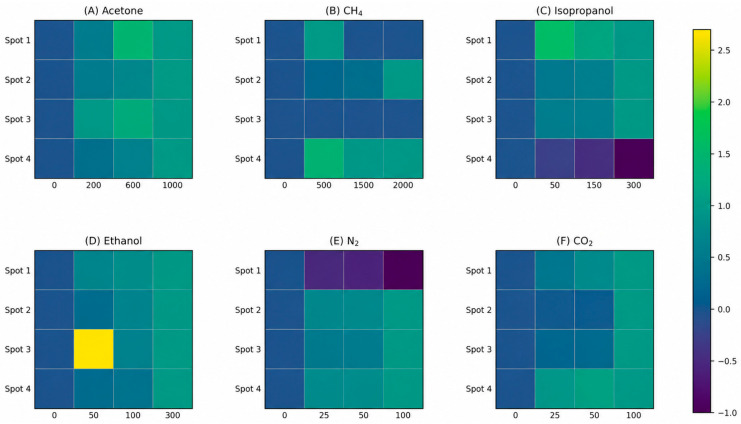
Heatmap representation of the normalized sensor responses for the investigated analytes: (**A**) acetone, (**B**) CH_4_, (**C**) isopropanol, (**D**) ethanol, (**E**) N_2_, and (**F**) CO_2_. In each panel, rows correspond to sensing spots and columns correspond to analyte concentrations, including the zero-concentration reference. The color scale represents the magnitude of the normalized response.

The MOSPR data recorded for the selected VOCs and gases are presented in Figure 3. Each spot has a different behavior when exposed to different gases. Recorded values show sensitivity comparable with similar approaches [2,25,26]; however, the materials employed in our approach are more accessible and easier to produce and use. The observed response patterns support the use of accessible and easily prepared sensing materials for generating analyte-dependent fingerprints in a multi-spot MOSPR/SPR assay. The sensing spots were designed as partially selective elements with different compositions, rather than as individually specific receptors. Consequently, analyte identification relies on the differential response pattern generated across the spot array and the MOSPR/SPR readout modes. This behavior is consistent with sensor-array and electronic-nose approaches, where selectivity emerges from the collective multivariate response. The heatmap representation illustrates these analyte-dependent fingerprints.

Analyte-specific calibration curves were used for concentration estimation and calculation of the limit of detection. The *LOD* was calculated according to

$$LOD=\frac{3.3\;s_{y/x}}{S}$$

where *s_y/x_* is the residual standard deviation of the linear regression and *S* is the slope of the selected response–concentration relationship. The resulting preliminary *LOD* estimates are presented in Table 1. Because several analytes were evaluated using only three concentration levels and the analytical performance has not yet been fully validated, the calibration curves and *LOD* values should be regarded as preliminary.

Although some calibration series comprised only three concentration levels, the corresponding responses showed a monotonic variation and a high coefficient of determination over the investigated range. For the present proof-of-concept study, these criteria were considered sufficient to demonstrate the existence of an approximately linear concentration-dependent response and to enable a preliminary comparison between sensing channels. Nevertheless, the resulting regression parameters and *LOD* values should be regarded as indicative rather than fully validated analytical figures of merit, and future studies should include additional concentration levels, particularly near the lower end of the calibration range.

**Figure 4:**
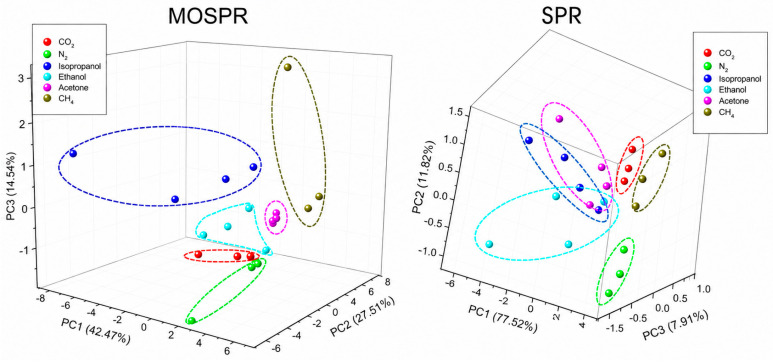
PCA score plots obtained from the extracted sensor-response features for the MOSPR-only and SPR-only feature sets. The first three principal components explained 80.56% of the variance for the MOSPR-only feature set and 97.73% of the variance for the SPR-only feature set. The percentage of variance explained by each individual principal component is indicated on the corresponding axis. Ellipses are included only as visual guides to indicate the dispersion of each analyte class in the PCA score space.

The multivariate structure of the multi-spot sensor responses was initially investigated using principal component analysis (PCA). The data were processed using the selected normalization procedure, and the covariance matrix of the resulting feature matrix was calculated. SPR and MOSPR datasets were first analyzed separately to allow direct comparison between the two detection modalities. The first three principal components accounted for 97.73% of the total variance in the SPR dataset and 80.56% in the MOSPR dataset. The corresponding three-dimensional score plots are presented in Figure 4. The MOSPR score plot showed a clearer separation of the analyte populations and more compact clustering of the individual gases and VOCs. In contrast, the SPR score plot exhibited partial overlap, particularly at low concentrations and among chemically related organic compounds such as acetone, ethanol, and isopropanol. These results suggest that the MOSPR measurements provide a more discriminative multivariate response under the investigated experimental conditions. This improved discrimination is attributed to the enhanced optical response generated by the magneto-plasmonic layers of the sensing chip and is consistent with the previously reported signal-to-noise behavior and sensitivity gain of the Au-Co MOSPR platform relative to classical SPR [16], together with the contribution of the magneto-plasmonic and gas-sensitive materials incorporated into the active sensing spots. The first three components of PCA of the combined MOSPR/SPR explained 75.43% of the total variance. The lower cumulative variance compared with the separate SPR and MOSPR analyses reflects the greater dimensional complexity and complementary information contained in the combined dataset rather than a loss of analytical information. Thus, PCA was used only as an exploratory and visualization tool, confirming that most of the relevant information can be represented in a reduced feature space, while still retaining the multivariate fingerprint generated by the active sensing spots.

To complement the exploratory PCA, the combined MOSPR and SPR responses were subsequently evaluated using a supervised shrinkage linear discriminant analysis (LDA) model. The supervised model used the analyte labels to determine discriminant directions that maximize separation between classes. Its performance was evaluated using leave-one-block-out cross-validation, in which all replicates belonging to the same experimental concentration or injection block were excluded together from model training. This validation strategy reduces information leakage between training and test data and provides a more realistic estimate of the classification performance for independent measurements. Although the complete multi-spot response pattern is required for reliable analyte identification, quantitative determination can subsequently be performed using the sensing spot and signal channel that exhibit the most suitable monotonic or linear response for the identified analyte. To prevent overly optimistic estimates caused by similarities among replicates from the same injection or concentration block, all replicates belonging to one block were excluded together during cross-validation. Group-preserving validation reduces information leakage between the training and test sets and provides a more realistic estimate of model generalization [27,28]. Shrinkage LDA was selected as an interpretable and regularized supervised classifier suitable for the present dataset, which contains correlated sensor features and a limited number of independent analyte concentration blocks. Nonlinear classifiers such as support vector machines or random forests may be useful for future expanded datasets, but were not used here in order to avoid overfitting and overly optimistic performance estimates on the current limited dataset.

Before model fitting, each sensor feature was robustly scaled within the corresponding training fold by subtracting the training-set median and dividing by the training-set interquartile range.

$$x_{ij}^{*}=\frac{x_{ij}-{median}_{train}(x_{j})}{{IQR}_{train}(x_{j})}$$

where *Q*_0.25_ and *Q*_0.75_ are the 25th and 75th percentiles, respectively ${IQR}_{train}\left(x_{j}\right)=Q_{0.75}\left(x_{j}\right)-Q_{0.25}(x_{j})$. Both *median_train_* and *IQR_train_* are calculated exclusively from the training data within each cross-validation fold to avoid data leakage.

The same scaling parameters were subsequently applied to the withheld test block. The complete dataset contains replicate measurements for six analytes recorded using 16 sensor-response variables. These variables corresponded to four sensing spots measured in four modes: MOSPR minimum, MOSPR reflectivity, SPR minimum, and SPR reflectivity. Each analyte–concentration pair was treated as a separate experimental group. Thus, all replicate measurements acquired for one analyte at one concentration received the same group identifier. This grouping was subsequently used during cross-validation. For each cross-validation iteration, one complete analyte–concentration group was removed from the dataset and used as the test set. All remaining groups were used as the training set. Consequently, replicate measurements originating from the same experimental block were never divided between training and testing. Within each iteration, the 16 sensor variables in the training set were robustly scaled. For each feature, the median of the training data was subtracted, and the result was divided by the interquartile range. The same median and interquartile-range values calculated from the training set were then used to transform the withheld test group. No preprocessing parameters were calculated using the test data. A linear discriminant analysis model with automatic covariance shrinkage was then fitted to the scaled training data, providing a more stable covariance estimate for correlated features and a limited number of independent blocks [29,30]. The model used the known analyte identity of the training measurements to estimate linear combinations of the sensor variables that maximized the separation among the six analyte classes. The fitted model was subsequently used to predict the analyte identity of every replicate in the withheld concentration block. This procedure was repeated until each analyte–concentration block had served once as the test set. The predictions obtained from all iterations were combined and compared with the known analyte labels. Overall accuracy was calculated as the fraction of correctly classified measurements, while balanced accuracy was calculated as the mean recall across the six analyte classes. The complete procedure was repeated for five different feature sets containing: all 16 MOSPR and SPR features; the eight MOSPR features; the eight SPR features; the eight minimum-mode features; and the eight reflectivity-mode features.

The best result was obtained using all 16 features. In this configuration, 68 of the 77 replicate measurements were correctly classified, corresponding to an overall accuracy of 88.3% and a balanced accuracy of 87.6%.

All CO_2_, N_2_, and CH_4_ measurements were classified correctly. Among the organic compounds, 12 of 13 acetone measurements, 8 of 12 ethanol measurements, and 8 of 12 isopropanol measurements were correctly assigned. The remaining errors occurred mainly between ethanol and isopropanol and, to a lesser extent, between ethanol and acetone (Table 2). This behavior is expected, since these volatile organic compounds have partially similar chemical properties and can produce overlapping responses on polymeric and oxide-based sensing layers.

The updated data-processing strategy confirms that the multi-spot MOSPR/SPR platform is capable of discriminating gases and VOCs at room temperature with high accuracy. Compared with PCA-only analysis, the supervised robust pipeline provides a quantitative performance estimate and demonstrates that the combined MOSPR + SPR feature set offers the strongest classification capability. The results support the use of MOSPR not only as a sensitivity-enhancing optical technique, but also as a source of complementary multivariate information for VOC identification in sensor-array applications. It should be noted that PCA and shrinkage LDA address different questions. PCA is an unsupervised method and identifies directions of maximum overall variance without using analyte-class labels. Therefore, the partial overlap observed in the SPR-only PCA projection should be interpreted as a limitation of unsupervised low-dimensional visualization rather than as a contradiction of the supervised classification results. By contrast, shrinkage LDA uses class information and identifies discriminant directions that maximize separation between analyte classes while regularizing the covariance estimate. The improved performance obtained with the combined MOSPR/SPR feature set therefore indicates that MOSPR and SPR responses provide complementary information that is better exploited by the supervised classifier than by visual inspection of the first principal components alone.

## 4. Conclusions

In summary, in this work, we demonstrate that using simple and accessible materials, one can discriminate selected gases and VOCs by MOSPR measurements at room temperature, with high impact and economic value. The materials used in our study are cost-effective and easily available, making the technology both practical and scalable. This technique leverages the magneto-plasmonic characteristics of the embedded materials within the sensing spots and the sensor chip itself. The unique combination of these materials contributes to improved sensitivity and analyte discrimination of the detection process. The improved robust supervised processing pipeline increased the analytical value of the multi-spot MOSPR/SPR assay by converting the sensor responses from a qualitative PCA-based fingerprint into a classification model, achieving 88.3% accuracy and 87.6% balanced accuracy under leave-one-block-out validation. These results support the further development of the MOSPR/SPR multi-spot platform toward environmental and industrial gas/VOC monitoring. However, application-level use will require additional validation, including dynamic response and recovery measurements, repeated exposure cycles, humidity and background-gas control, long-term stability testing, and evaluation under field-relevant conditions.

## Figures and Tables

**Table 1:** Preliminary *LOD* estimates obtained for the selected VOCs and gases.

Analyte	Selected Channel	R^2^	LOD	Unit
Acetone	MO min spot 1	0.9947	131	ppm
Methane	SPR refl spot 2	0.8936	1230	ppm
Ethanol	SPR min spot 4	0.9998	25	ppm
Isopropanol	MO min spot 2	0.9992	48	ppm
CO_2_	SPR refl spot 1	0.9993	4.8	%
N_2_	MO refl spot 4	0.9999	1.9	%

**Table 2:** Confusion matrix for the complete MOSPR + SPR model.

Actual/Predicted	Acetone	CH_4_	CO_2_	Ethanol	Isopropanol	N_2_
Acetone	12	0	0	1	0	0
CH_4_	0	10	0	0	0	0
CO_2_	0	0	15	0	0	0
Ethanol	3	0	0	8	1	0
Isopropanol	0	0	0	4	8	0
N_2_	0	0	0	0	0	15

## Data Availability

Data are available from the corresponding author upon reasonable request.
